# The correlation between physical exercise and cognitive function in older adults with cognitive impairment mediated by inhibitory control and possible cognitive processing mechanisms

**DOI:** 10.3389/fpubh.2026.1818047

**Published:** 2026-05-20

**Authors:** Bingbin Xie, Fuhai Wang, Simin Xu, Chen Wei, Tao Meng

**Affiliations:** 1 Department of Physical Education, East China University of Political Science and Law, Shanghai, China; 2 College of Sports Science, Shenyang Normal University, Shenyang, China; 3 Department of Physical Education, Shanghai University of Sport, Shanghai, China; 4 Department of Sports and Health, Shanghai Lixin University of Accounting and Finance, Shanghai, China

**Keywords:** cognitive function, cognitive impairment, inhibitory control, older adults, physical exercise

## Abstract

**Purpose:**

Cognitive impairment is currently a prominent age-related challenge among older adults. This study examined the relationships between inhibitory control, cognitive function, EEG indicators, and physical exercise in older adults with cognitive impairment, and explored the mediating roles of EEG indicators and inhibitory control in the association between physical exercise and cognitive function.

**Patients and methods:**

A total of 239 older adults were recruited, of whom 209 were included in the final analysis. Cognitive function and physical exercise were assessed using the Montreal Cognitive Assessment (MoCA) and Physical Activity Rating Scale-3 (PARS-3), respectively. Inhibitory control was evaluated using the Stroop task and 5 min of resting-state EEG signals were recorded.

**Results:**

Cognitive function was significantly associated with inhibitory control in older adults with cognitive impairment. *θ*(FP1 + FP2) power emerged as a specific EEG indicator correlated with both cognitive function and inhibitory control. A significant negative correlation was observed between physical exercise volume and resting-state *θ*(FP1 + FP2) power (*r* = −0.288, *p* < 0.05). Mediation analysis revealed a significant direct association between physical exercise and cognitive function (*B* = 0.037, 95%CI: 0.005, 0.063, *p* = 0.020). Furthermore, physical exercise was indirectly associated with cognitive function through three significant pathways: the independent mediation of *θ*(FP1 + FP2) power (*B* = 0.008, *p* = 0.022), the independent mediation of inhibitory control (*B* = 0.013, *p* = 0.011), and a chain mediation from *θ*(FP1 + FP2) power to inhibitory control (*B* = 0.003, 95%CI: 0.001, 0.009, *p* = 0.028). Collectively, these indirect pathways accounted for 39.0% of the total association.

**Conclusion:**

Higher volumes of physical exercise in older adults are associated with superior inhibitory control and cognitive performance, as well as lower resting-state *θ*(FP1 + FP2) power. *θ*(FP1 + FP2) serves as a shared neuroelectrophysiological marker linked to both inhibitory control and cognitive function. Furthermore, exploratory SEM suggested that this EEG-specific indicator and inhibitory control may statistically mediate the association between physical exercise and cognitive function.

## 1 Introduction

As global life expectancy continues to rise, population aging has emerged as a pervasive challenge with profound societal implications (1). It is projected that the global population aged 65 and older will reach 1.5 billion by 2050 (2), the incidence of age-related degenerative diseases is steadily increasing (3), with cognitive impairment being one of the most prominent conditions (4), affecting up to 33.59% of the older adult population (5). A critical feature of this cognitive decline is the parallel deterioration of inhibitory control (6, 7). As a core executive function, inhibitory control refers to the ability to suppress attention toward incongruent or task-irrelevant stimuli (8). Its reduction renders older adults highly susceptible to external interference, leading to significant attention deficits and cognitive disruption (9). Nevertheless, despite age-related declines, inhibitory control retains a degree of neuroplasticity throughout the aging process (10).

Physical exercise, a widely accessible non-pharmacological intervention (11–13), has garnered significant attention for its potential to delay cognitive decline. The World Health Organization recommends that older adults engage in at least 150 min of moderate-intensity or 75 min of vigorous-intensity physical activity per week (14). Research indicates that older adults with low levels of physical exercise exhibit a greater degree of brain atrophy (15), and a sedentary lifestyle among older adults is associated with poorer cognitive function (16). Furthermore, a lack of physical exercise is highly prevalent among older adults with cognitive impairment (17, 18). Conversely, cross-sectional evidence indicates (19, 20) that regular physical exercise is associated with a reduced incidence of cognitive impairment, while experimental studies (10, 21, 22) confirm its efficacy in enhancing both overall cognition and specific inhibitory control. Mechanistically, physical activity is linked to improved brain health by facilitating neuroplasticity (23), stimulating neuronal regeneration, and upregulating neurotrophic factors (24, 25). Furthermore, long-term exercise helps mitigate neurodegenerative structural changes, strengthens functional connectivity, and optimizes prefrontal network efficiency, ultimately supporting better attention regulation and inhibitory control in older adults (26, 27).

Electroencephalography (EEG) provides a low-cost, non-invasive method to measure cortical neural electrical activity (28, 29). It offers unique advantages over traditional neuropsychological scales and structural imaging due to its high temporal resolution and sensitivity to dynamic cognitive processing (30–32). While *α* and *β* rhythms often serve as indicators for predicting cognitive performance in healthy adults (33), individuals with cognitive impairment characteristically exhibit a pathological shift towards increased low-frequency power (*θ*, *δ*) and decreased high-frequency activity (*α*, *β*) compared to their healthy counterparts (34–36).

Previous studies have examined the associations among physical function, EEG characteristics, and cognitive performance in older adults with cognitive impairment (29, 37–40). However, relatively limited evidence has focused specifically on inhibitory control as a behavioral pathway linking physical exercise to global cognitive function, particularly when combined with resting-state EEG indicators. Therefore, the present study examined whether resting-state EEG activity and inhibitory control jointly explain the association between physical exercise and cognitive function in older adults with cognitive impairment. Based on the above evidence, we proposed the following directional hypotheses: H1: Higher physical exercise volume would be associated with better global cognitive function and better inhibitory control performance in older adults with cognitive impairment. H2: Higher levels of the EEG-specific indicator would be associated with poorer cognitive function and poorer inhibitory control performance. H3: The EEG-specific indicator and inhibitory control would statistically mediate the association between physical exercise volume and cognitive function. H4: Older adults with moderate or high levels of physical exercise would show better cognitive function, better inhibitory control, and lower levels of the EEG-specific indicator than those with low physical exercise levels.

## 2 Methods

### 2.1 Participant recruitment

An *a priori* statistical power analysis was conducted using G*Power 3.1 software. The anticipated effect size was set at a medium level (Cohen’s *f*^2^ = 0.15), with a significance level of *α* = 0.05, a target statistical power of 1−*β* = 0.80, and a maximum of 3 predictor variables. The results indicated that a minimum sample size of 77 participants is required to satisfy these testing conditions.

**Figure 1:**
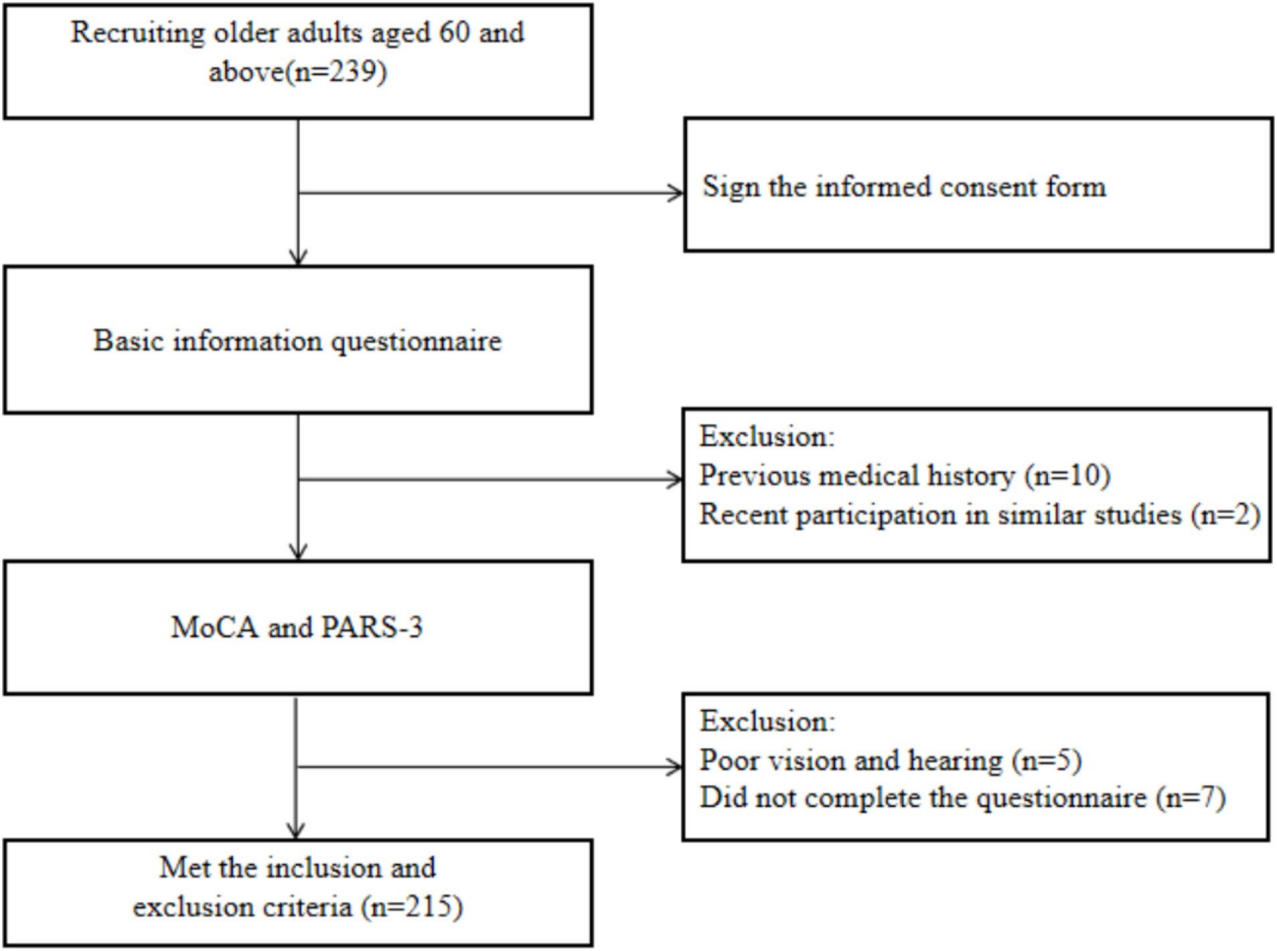
Recruitment process.

The study protocol was approved by the Ethics Committee of Shanghai University of Sport (Approval No. 102772020RT060). Written informed consent was obtained from all participants prior to their enrollment in the study. Between June and August 2024, a total of 239 older adults (>60 years of age) were recruited via convenience sampling from four senior care centers in Shanghai. Recruitment was conducted on a strictly voluntary basis through localized health seminars and promotional posters. The detailed recruitment flowchart is illustrated in Figure 1.

### 2.2 Inclusion criteria

Participants were eligible for inclusion if they: (1) were aged 60 years or older; (2) were right-handed; (3) possessed normal or corrected-to-normal vision and hearing; and (4) exhibited a stable mental status with the capacity for normal verbal communication.

### 2.3 Exclusion criteria

Individuals were excluded from the study if they met any of the following criteria: (1) presence of severe cardiovascular diseases or major organic pathologies; (2) diagnosis of severe musculoskeletal disorders, Parkinson’s disease, or any other medical conditions significantly impairing motor function, gait, or standing balance; (3) current or recent use of psychotropic medications, cholinesterase inhibitors, or other pharmacological agents known to influence cognitive or physical performance; or (4) recent participation in similar clinical or behavioral trials, to prevent potential practice effects and data confounding.

### 2.4 Testing procedure

**Figure 2:**
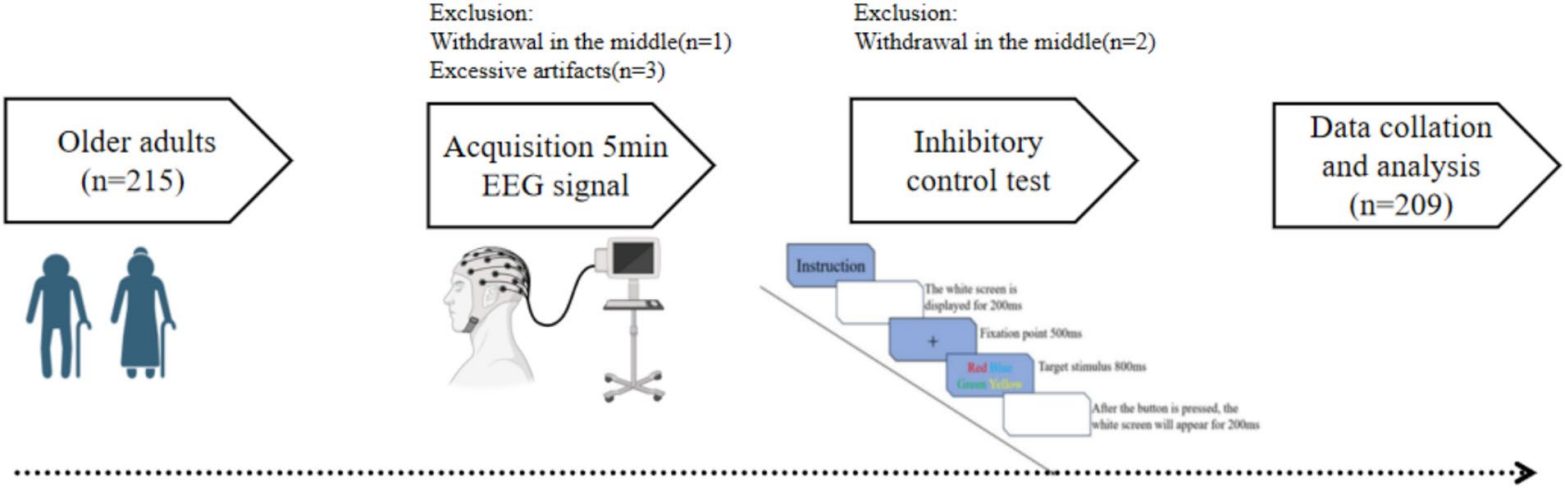
Participant flow chart.

All experimental sessions were scheduled daily between 13:30 and 16:30 to control for circadian variations. Participants completed two laboratory visits. During the first visit, the research team provided a detailed explanation of the experimental protocol. Participants then provided their basic demographic information and completed the Montreal Cognitive Assessment (MoCA) and the Physical Activity Rating Scale-3 (PARS-3). On the second visit, participants underwent resting-state EEG signal recording followed by the Stroop task. The comprehensive testing procedure is illustrated in Figure 2. To minimize confounding physiological factors, participants were instructed to refrain from strenuous physical exercise, avoid consuming any caffeinated or alcoholic beverages for 24 h prior to the test, and maintain a regular sleep schedule.

### 2.5 Testing tools

#### 2.5.1 Demographic and health history questionnaire

The demographic and health data collected included the participants’ names, ages, heights, weights, marital statuses, educational levels, lifestyle habits (e.g., smoking, alcohol consumption, and dietary patterns), and history of falls.

#### 2.5.2 MoCA Scale

The MoCA is a widely validated instrument used to assess global cognitive function in older adults. It evaluates multiple cognitive domains, including visuospatial/executive function, naming, memory, attention, language fluency, abstract thinking, delayed recall, and orientation. The maximum score is 30, with higher scores indicating superior cognitive performance. An established cut-off score of <26 points was used to identify cognitive impairment. To adjust for educational bias, two additional points were awarded to participants with ≤6 years of formal education, and one point was awarded to those with 6–12 years of education (no adjustment was made for >12 years). This study utilized the Beijing version of the MoCA, which has demonstrated excellent test–retest reliability (*r* = 0.898) (41). Based on criteria from previous research (42), participants were stratified into a mild cognitive impairment group (20–25 points) and a moderate-to-severe cognitive impairment group (≤19 points).

#### 2.5.3 PARS-3 Scale

Habitual physical activity was evaluated using the PARS-3, originally developed by Hashimoto and later revised for the Chinese population by Liang et al. (43). This scale assesses physical exercise across three dimensions: intensity (1–5 points), duration (0–4 points), and frequency (1–5 points). The total score is calculated as the product of these three dimensions (Intensity × Duration × Frequency), yielding a continuous score ranging from 0 to 100. Based on established norms, physical activity levels were categorized as low (≤ 19 points), moderate (20–42 points), or high (≥ 43 points). The scale exhibited strong internal consistency (Cronbach’s *α* = 0.856) and test–retest reliability (*r* = 0.82), demonstrating robust psychometric properties in this population (44).

#### 2.5.4 Inhibitory control test

Inhibitory control was evaluated using a computerized Stroop color-word task. The paradigm comprised two blocks, each consisting of a practice phase followed by a formal experimental phase. The practice phase included 20 trials that provided immediate visual feedback regarding response accuracy and reaction time. Participants were required to achieve a minimum accuracy rate of 75% to proceed to the formal experiment; those failing to meet this criterion were permitted up to three practice attempts. Following a 30-s rest period, the formal testing commenced.

The visual stimuli consisted of four Chinese color words (“red,” “yellow,” “blue,” and “green”) presented in four ink colors (red, yellow, blue, and green). Following the presentation of a central fixation cross (“+”), the target stimulus appeared in the center of the screen. The stimuli were presented in a randomized order with consistent font sizes. The formal experiment was divided into a congruent condition block and an incongruent condition block. In the congruent block (Block 1; 48 trials), the semantic meaning of the word matched its ink color. Participants were instructed to identify the ink color by pressing designated keys on a standard keyboard (Red→ “D,” Yellow → “F,” Blue → “J,” Green → “K”). In the incongruent block (Block 2; 96 trials), the semantic meaning and the ink color mismatched. Participants were instructed to suppress the automatic word-reading response and strictly identify the ink color using the same key-mapping. All stimuli were programmed and presented using E-Prime 3.0.9 software. The primary behavioral indices extracted for inhibitory control assessment were the congruent condition accuracy rate (CAR), congruent condition reaction time (CRT), incongruent condition accuracy rate (IAR), and incongruent condition reaction time (IRT).

### 2.6 EEG signal acquisition and processing

All EEG recordings were conducted in a sound-attenuated, electromagnetically shielded, and well-ventilated laboratory with controlled temperature and humidity. Prior to the experiment, participants were instructed to wash and dry their hair to ensure optimal scalp conductance. During the recording, participants were seated in a comfortable armchair. They were instructed to keep their eyes closed, remain relaxed yet awake, and actively minimize ocular and muscular artifacts (e.g., blinking, jaw clenching, swallowing, and gross bodily movements).

**Figure 3:**
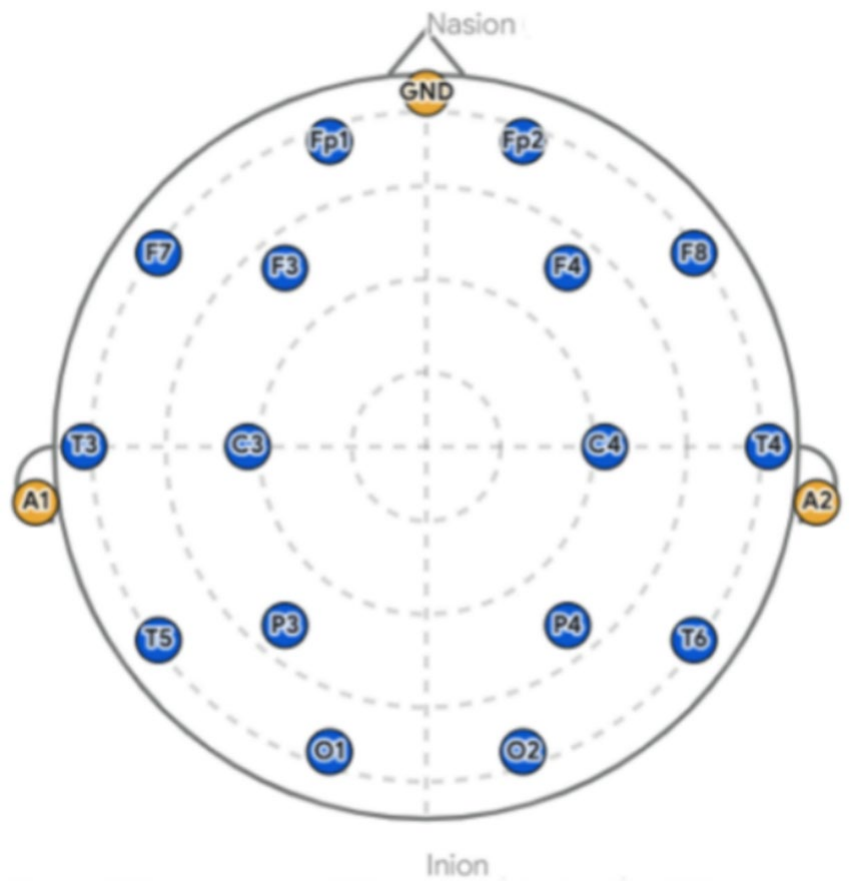
Electrode distribution diagram.

EEG signals were acquired using an NCERP-190012 electroencephalography system (Shanghai Nuo Cheng Electric Co., Ltd., Shanghai, China). Sixteen electrodes were positioned according to the international 10–20 system (Fp1, Fp2, F3, F4, F7, F8, C3, C4, P3, P4, O1, O2, T3, T4, T5, and T6). The electrode distribution diagram is shown in Figure 3. The ground electrode (GND) was placed at the midline of the forehead, and the physical reference electrodes were positioned on the bilateral mastoids (A1, A2). Electrode-skin impedance was strictly maintained below 5 kΩ. The EEG signals were amplified, digitized at a sampling rate of 500 Hz, and online band-pass filtered between 0.3 and 30 Hz.

EEG data preprocessing was performed using MATLAB R2023b and the EEGLAB 2024.0 toolbox. The preprocessing pipeline comprised the following steps: (1) importing raw continuous EEG data and assigning channel locations; (2) detecting and spherically interpolating bad channels; (3) re-referencing the data to the common average reference; (4) manually rejecting segments with severe baseline drift; (5) downsampling the data to 256 Hz; and (6) segmenting the continuous data into 2-s, non-overlapping epochs. To address non-physiological noise, Infomax Independent Component Analysis (ICA) was applied. Artifactual components (e.g., ocular, muscular, and cardiac artifacts) were manually identified and rejected based on scalp topography, power spectra, and time-domain characteristics. Subsequently, anomalous epochs with amplitude fluctuations exceeding ±100 μV were automatically rejected. For spectral analysis, a Fast Fourier Transform (FFT) was performed on the artifact-free epochs to calculate the absolute power values for each electrode. The data were decomposed into six canonical frequency bands: *δ* (1–4 Hz), *θ* (4–8 Hz), *α*1 (8–10.5 Hz), *α*2 (10.5–13 Hz), *β*1 (13–20 Hz), and *β*2 (20–30 Hz). The absolute power values of symmetrical hemispheric electrodes were aggregated by brain region (P_left_ + P_right_), and the processed data were exported for subsequent statistical analysis.

### 2.7 Statistical analyses

Statistical analyses were performed using SPSS version 29.0 and Amos version 24.0. The normality of all continuous variables—including MoCA scores, PARS-3 scores, the four Stroop task indices, and absolute EEG power values across distinct frequency bands and brain regions—was systematically assessed using the Shapiro–Wilk test, supplemented by visual inspection of histograms and *Q*-*Q* plots. Normally distributed continuous variables are expressed as the mean ± standard deviation (SD), with between-group comparisons conducted using parametric tests (e.g., one-way ANOVA) and relationships evaluated via Pearson correlation coefficients. Non-normally distributed variables are presented as the median and interquartile range (IQR), and were analyzed using non-parametric alternatives, with relationships assessed via Spearman’s rank-order correlations. Categorical variables are reported as frequencies (percentages) and were compared using the Chi-square (*χ*^2^) test. To evaluate differences in continuous variables among the three physical activity groups while rigorously controlling for potential confounding factors, a one-way analysis of covariance (ANCOVA) was employed. Significant main effects were further investigated using post-hoc pairwise comparisons with Bonferroni correction. Furthermore, to address the risk of Type I errors associated with multiple comparisons (particularly for EEG metrics), two-sided *p*-values were adjusted using the Benjamini–Hochberg procedure to control the false discovery rate (FDR) (45). Results were considered statistically significant when the adjusted P ≤ P_FDR_. Structural equation modeling (SEM) was conducted using Amos 24.0 to examine the hypothesized statistical mediation pathway among physical exercise, *θ*(FP1 + FP2) power, inhibitory control, and cognitive function. Physical exercise was entered as the independent variable, cognitive function as the dependent variable, and *θ*(FP1 + FP2) power and inhibitory control as mediating variables. Sex and educational attainment were included as covariates because they may be associated with cognitive performance and physical exercise behavior in older adults. All continuous variables were standardized prior to model estimation. Model fit was evaluated using CMIN/df, RMR, RMSEA, GFI, NFI, IFI, and CFI. Path coefficients and indirect effects were estimated using a non-parametric bootstrap procedure with 5,000 resamples. A mediation effect was considered statistically significant when the 95% confidence interval did not include zero. Given the cross-sectional design, the SEM was interpreted as an exploratory statistical mediation model rather than as evidence of causal pathways.

## 3 Results

### 3.1 Data quality inspection

Initially, a total of 239 participants were recruited for this study. During the data acquisition and preprocessing stages, 30 participants were excluded. To eliminate potential systematic biases arising from sample attrition, an attrition analysis (missing data analysis) was conducted, comparing the participants included in the final analysis (*n* = 209) with those who were excluded (*n* = 30). The results revealed no statistically significant differences between the two groups in terms of age, height, or weight (all *p* > 0.05). These findings suggest that the missing data mechanism in this study aligns with Missing Completely At Random (MCAR). Given that the excluded data primarily stemmed from severe contamination of raw physiological signals or invalid behavioral responses—rendering statistical imputation neither feasible nor appropriate—this study performed the primary statistical inferences using the complete dataset under the MCAR assumption to ensure the robustness of the results.

### 3.2 Basic information

A total of 209 participants (88 males and 121 females) with a mean age of 70.99 ± 7.49 years were included in this study. The distribution of MoCA scores in the present study ranged from 6 to 25, with a mean of 17.98 ± 4.78, a median of 18, and an IQR of 15–22. The score distribution followed an approximately normal distribution, exhibiting no significant ceiling or floor effects. Baseline characteristics were compared between the mild cognitive impairment group and the moderate-to-severe cognitive impairment group. The analysis revealed statistically significant differences in age, educational level, and marital status (all *p* < 0.05). However, no significant differences were observed across other demographic variables (all *p* > 0.05), as detailed in Table 1. These findings indicate that the severity of cognitive impairment in older adults is significantly associated with age, educational attainment, and marital status. Notably, the likelihood of presenting with moderate-to-severe cognitive impairment is higher among older individuals and those with lower educational levels.

**Table 1:** Basic information table.

Characteristics	Total (*n* = 209)	Mild cognitive impairment (*n* = 118)	Moderate-to-severe cognitive impairment (*n* = 91)	Difference test
Age	70.99 ± 7.493	69.23 ± 6.586	73.27 ± 8.001	*t* = −3.909, *p*<0.001
Gender (%)				
Male	88 (42.1)	51 (43.2)	37 (40.7)	*χ*^2^ = 0.138, *p* = 0.710
Female	121 (57.9)	67 (56.8)	54 (59.3)	
Height	1.611 ± 0.073	1.614 ± 0.065	1.607 ± 0.083	*t* = 0.712, *p* = 0.477
Weight	61.888 ± 9.34	62.911 ± 9.247	60.560 ± 9.363	*t* = 1.812, *p* = 0.071
Education level (%)				
Illiteracy	35 (16.7)	9 (7.6)	26 (28.6)	*χ*^2^ = 26.708, *p*<0.001
Primary school	79 (37.8)	37 (33.1)	42 (44.0)	
Junior high school	61 (29.2)	46 (37.3)	15 (18.7)	
Senior middle school and over	34 (16.3)	26 (22.0)	8 (3.8)	
Smoking (%)				
Yes	52 (24.9)	31 (26.3)	21 (23.1)	*χ*^2^ = 0.280, *p* = 0.596
No	157 (75.1)	87 (73.7)	70 (76.9)	
Drinking (%)				
Yes	60 (28.7)	38 (32.2)	22 (24.2)	*χ*^2^ = 1.618, *p* = 0.203
No	149 (71.3)	80 (67.8)	69 (75.8)	
Dietary habits (%)				
Vegetarian	52 (24.9)	28 (23.7)	24 (26.4)	*χ*^2^ = 0.328, *p* = 0.849
Meat-based diet	6 (2.9)	3 (2.5)	3 (3.3)	
Mixed diet	151 (72.2)	87 (73.7)	64 (70.3)	

### 3.3 Differences in inhibitory control among older adults with varying degrees of cognitive impairment

Given that the behavioral data did not satisfy the assumption of normality, Mann–Whitney *U* tests were employed to compare Stroop task performance between older adults with mild and moderate-to-severe cognitive impairment (Table 2). Results indicated significant intergroup differences across all inhibitory control indices. Specifically, the mild cognitive impairment group exhibited a significantly higher accuracy rate in the congruent condition compared to the moderate-to-severe group (*Z* = −6.689, *p* < 0.001, *r* = 0.46), alongside significantly shorter reaction times (*Z* = −4.222, *p* < 0.001, *r* = 0.29). Under the incongruent condition, the mild group maintained a significantly superior accuracy rate (*Z* = −3.388, *p* < 0.001, *r* = 0.23) and significantly faster reaction times than the moderate-to-severe group (*Z* = −2.695, *p* = 0.007, *r* = 0.19). These findings suggest that a lower degree of cognitive impairment is significantly associated with superior inhibitory control capacity.

**Table 2:** Differences in inhibitory control between older adults with cognitive impairment.

Indicators	Mild cognitive impairment (*n* = 118)	Moderate-to-severe cognitive impairment (*n* = 91)	*Z*	*p*	*r*
CAR	0.996 (0.917, 1.000)	0.780 (0.500, 0.969)	−6.689	<0.001	0.46
CRT	0.998 (0.904, 1.157)	1.149 (0.987, 1.254)	−4.222	<0.001	0.29
IAR	0.941 (0.667, 1.000)	0.750 (0.390, 0.972)	−3.388	<0.001	0.23
IRT	1.112 (1.036, 1.184)	1.153 (1.058, 1.451)	−2.695	0.007	0.19

### 3.4 Relationship between inhibitory control, cognitive function, and physical exercise in older adults with cognitive impairment

**Figure 4:**
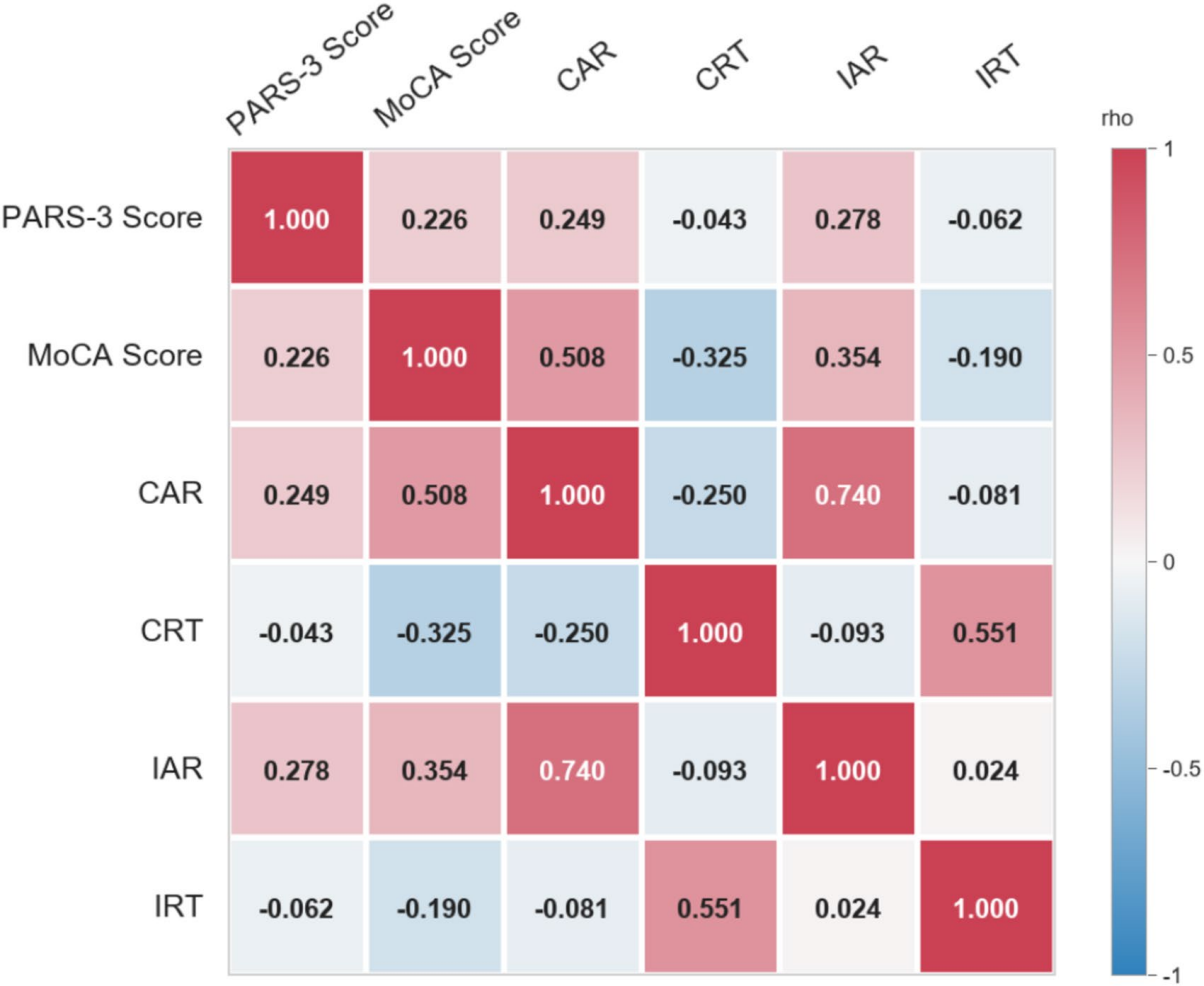
Correlation among inhibitory control, cognitive function, and physical exercise.

Spearman’s rank-order correlation analyses were conducted to examine the relationships between inhibitory control, cognitive function, and physical exercise volume. The results (Figure 4) demonstrated that both CAR (*r* = 0.508, *p* < 0.05) and IAR (*r* = 0.354, *p* < 0.05) were positively correlated with MoCA scores. Conversely, CRT (*r* = −0.325, *p* < 0.05) and IRT (*r* = −0.190, *p* < 0.05) exhibited negative correlations with MoCA scores. Furthermore, physical exercise volume was positively correlated with MoCA scores (*r* = 0.226, *p* < 0.05), CAR (*r* = 0.249, *p* < 0.05), and IAR (*r* = 0.278, *p* < 0.05). These findings indicate that higher volumes of physical exercise are significantly associated with both better inhibitory control and superior cognitive performance in older adults with cognitive impairment.

### 3.5 Relationship between inhibitory control, cognitive function, and EEG indicators across brain regions in older adults with cognitive impairment

Spearman’s rank correlations were similarly utilized to explore the relationships between inhibitory control, cognitive function, and whole-brain EEG indicators. All resulting *p*-values were subjected to FDR correction to strictly control for multiple comparisons. As presented in Table 3, the frontopolar *θ* power, denoted as *θ*(FP1 + FP2), exhibited significant negative correlations with MoCA scores, CAR, and IAR (all adjusted *p* < 0.05). Consequently, *θ*(FP1 + FP2) emerged as the unique EEG index jointly associated with both cognitive function and inhibitory control in this population, hereafter referred to as the specific EEG indicator. These results suggest that better cognitive function and inhibitory control capacities in cognitively impaired older adults are robustly correlated with lower resting-state *θ*(FP1 + FP2) power. The complete relevant data are in Supplementary material S1.

**Table 3:** The relationship between inhibitory control, cognitive function and EEG indicators in older adults with cognitive impairment.

EEG indicator	MoCA score			Inhibitory control											
				CAR		CRT				IAR			IRT		
	*r*	P_FDR_	*p*	*r*	P_FDR_	*p*	*r*	P_FDR_	*p*	*r*	P_FDR_	*p*	*r*	P_FDR_	*p*
*θ*(FP1 + FP2)	−0.269*	0.001	0.001	−0.279*	0.001	0.001	−0.058	0.042	0.404	−0.281*	0.002	0.001	0.000	0.050	0.998

* indicates *p* ≤ PFDR, indicating a significant correlation.

### 3.6 Relationship between physical exercise and EEG-specific indicators

**Figure 5:**
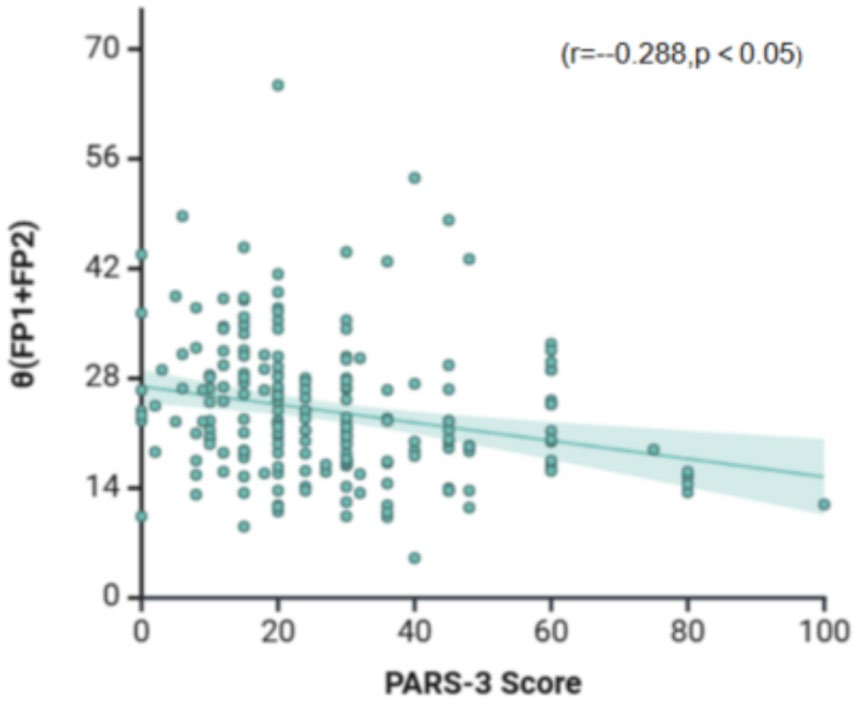
Relationship between physical exercise and the EEG-specific indicator.

To further identify specific EEG indicators sensitive to physical exercise volume, Spearman correlation analysis was performed after controlling for confounding variables. The results (Figure 5) revealed a significant negative correlation between *θ* (FP1 + FP2) power and PARS-3 scores (*r* = −0.288, *p* < 0.05). These findings suggest that *θ* (FP1 + FP2) is a specific EEG indicator associated with physical exercise volume, where higher levels of physical exercise in older adults with cognitive impairment are associated with lower *θ* (FP1 + FP2) power values.

### 3.7 Comparison of inhibitory control, cognitive function, and EEG-specific indicators in older adults with cognitive impairment based on physical exercise

**Figure 6:**
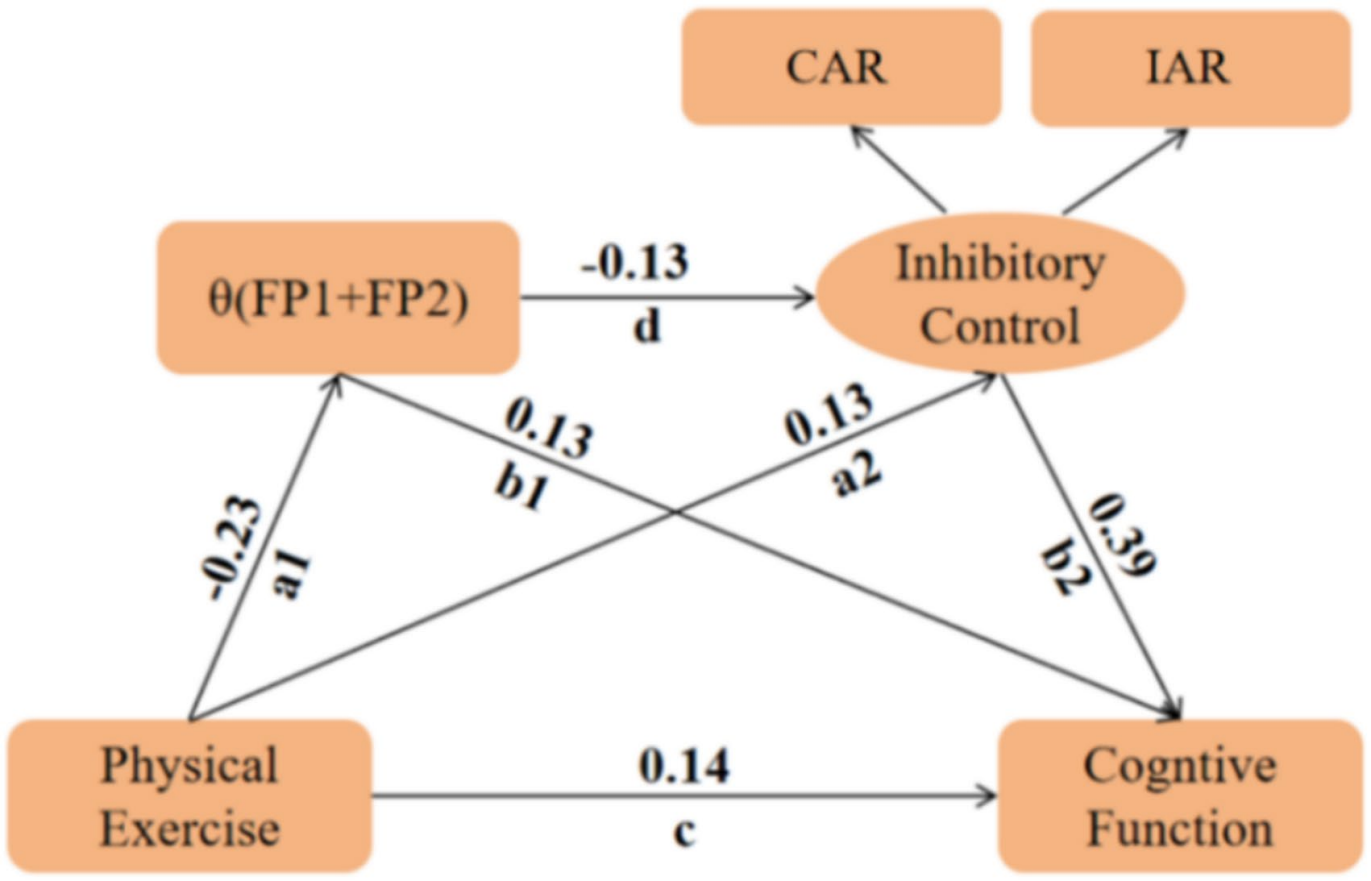
Pathway construction of physical exercise, inhibitory control, cognitive function, and EEG-specific indicators.

To investigate differences in cognitive function, inhibitory control, and the EEG-specific indicator across physical exercise levels, ANCOVA was conducted with sex and educational attainment controlled as covariates (Table 4). The results showed significant group differences in MoCA scores, *F*(2, 204) = 4.760, *p* = 0.010, partial *η*^2^ = 0.045. Bonferroni-adjusted *post-hoc* comparisons indicated that the high physical exercise group had significantly higher MoCA scores than the low physical exercise group (*p* = 0.010), whereas the differences between the low and moderate groups and between the moderate and high groups were not statistically significant. Significant group differences were also observed for CAR, *F*(2, 204) = 4.891, *p* = 0.008, partial *η*^2^ = 0.046. Both the moderate and high physical exercise groups showed significantly higher CAR than the low physical exercise group (*p* = 0.014 and *p* = 0.045, respectively), while no significant difference was found between the moderate and high groups. For IAR, the ANCOVA revealed a significant group difference, *F*(2, 204) = 6.187, *p* = 0.002, partial *η*^2^ = 0.057. Post-hoc comparisons showed that the moderate physical exercise group had significantly higher IAR than the low physical exercise group (*p* = 0.002), whereas the difference between the low and high groups did not reach statistical significance (*p* = 0.077). For the EEG-specific indicator, *θ*(FP1 + FP2) power differed significantly across physical exercise groups, *F*(2, 204) = 4.763, *p* = 0.010, partial *η*^2^ = 0.045. The low physical exercise group showed significantly higher *θ*(FP1 + FP2) power than both the moderate and high physical exercise groups (*p* = 0.048 and *p* = 0.016, respectively), with no significant difference between the moderate and high groups. These results suggest that, among older adults with cognitive impairment, higher levels of physical exercise are associated with superior inhibitory control and cognitive performance, as well as lower *θ*(FP1 + FP2) power values (Figure 6).

**Table 4:** Comparison of differences in inhibitory control, cognitive function and EEG-specific indicators among different levels of physical exercise.

Indicators	Physical exercise level (M ± SE)			Difference test		*Post-hoc* multiple comparisons			partial *η*^2^
	Low (*n* = 66)	Moderate (*n* = 103)	High (*n* = 40)	*F*	*p*	Low *Vs* Moderate	Low *Vs* High	Moderate *Vs* High	
MoCA	16.775 ± 0.525	18.245 ± 0.422	19.292 ± 0.675	4.760	0.010	0.093	0.010	0.578	0.045
CAR	0.771 ± 0.026	0.866 ± 0.021	0.874 ± 0.033	4.891	0.008	0.014	0.045	1.000	0.046
IAR	0.628 ± 0.037	0.794 ± 0.030	0.764 ± 0.048	6.187	0.002	0.002	0.077	1.000	0.057
*θ*(FP1 + FP2)	26.365 ± 1.064	23.031 ± 0.856	21.486 ± 1.370	4.763	0.010	0.048	0.016	1.000	0.045

### 3.8 Mediation analysis

Based on the established interrelationships among physical exercise, inhibitory control, the EEG-specific indicator, and cognitive function, a structural equation model (SEM) was constructed. In this model, physical exercise served as the independent variable, cognitive function as the dependent variable, and both the EEG-specific indicator and inhibitory control as mediating variables. Prior to model estimation, multicollinearity among all independent variables was assessed via linear regression. Diagnostic results (Table 5) showed that the Variance Inflation Factors (VIF) ranged from 1.066 to 2.259, well below the conservative threshold of 5.0, indicating the absence of significant multicollinearity and ensuring the stability of subsequent parameter estimates. After controlling for sex and educational attainment, the SEM showed the following fit indices: CMIN/df = 4.665, RMR = 0.084, GFI = 0.973, RMSEA = 0.133, NFI = 0.949, IFI = 0.960, and CFI = 0.957. The incremental fit indices, including GFI, NFI, IFI, and CFI, were all above 0.90, suggesting that the proposed structural framework was generally supported by the data (46, 47). However, the RMSEA value was elevated, indicating that the absolute fit of the model was suboptimal. Previous methodological research has noted that RMSEA may be less stable in models with small degrees of freedom (48). Therefore, the SEM results should be interpreted cautiously as exploratory statistical mediation findings. The mediation test results are presented in Table 6. The total association between physical exercise and cognitive function was significant, *B* = 0.061, 95% CI: 0.030–0.094, *p* = 0.001. The direct association was also significant, *B* = 0.037, 95% CI: 0.005–0.063, *p* = 0.020. The total indirect association was significant, *B* = 0.024, 95% CI: 0.009–0.045, *p* = 0.004, accounting for 39.0% of the total association. Specifically, *θ*(FP1 + FP2) power showed a significant indirect association, *B* = 0.008, 95% CI: 0.001–0.019, *p* = 0.022. Inhibitory control also showed a significant indirect association, *B* = 0.013, 95% CI: 0.003–0.028, *p* = 0.011. In addition, the serial pathway through *θ*(FP1 + FP2) power and inhibitory control was significant, *B* = 0.003, 95% CI: 0.001–0.009, *p* = 0.028. These findings suggest that *θ*(FP1 + FP2) power and inhibitory control may statistically mediate the association between physical exercise and cognitive function in older adults with cognitive impairment.

**Table 5:** Collinearity analysis.

Indicators	*B*	SE	Beta	Difference test		95%CI	Collinearity analysis	
				*t*	*p*		Tolerance	VIF
CAR	14.828	1.813	0.682	8.178	0.000	(11.254, 18.403)	0.443	2.259
IAR	−2.875	1.290	−0.186	−2.229	0.027	(−5.417, −0.332)	0.443	2.256
*θ*(FP1 + FP2)	−0.083	0.031	−0.152	−2.663	0.008	(−0.144, −0.022)	0.938	1.066

**Table 6:** Bootstrap test results of mediating associations.

Type of association	*B*	95%CI	P	SE	Proportion of association (%)
Total association	0.061	0.030, 0.094	0.001	0.016	100
Direct association	0.037	0.005, 0.063	0.020	0.015	60.66
Path a1-b1	0.008	0.001, 0.019	0.022	0.004	13.11
Path a2-b2	0.013	0.003, 0.028	0.011	0.006	21.31
Path a1-d-b2	0.003	0.001, 0.009	0.028	0.003	4.92

## 4 Discussion

Taken together, these findings generally supported H1 and H2 and provided partial support for H3 and H4. Specifically, physical exercise volume was positively associated with cognitive function and inhibitory control, whereas *θ*(FP1 + FP2) power was negatively associated with these outcomes. The exploratory covariate-adjusted SEM further suggested possible statistical mediation through *θ*(FP1 + FP2) power and inhibitory control. The present study demonstrates that higher volumes of physical exercise are significantly associated with superior inhibitory control and cognitive performance in older adults with cognitive impairment, aligning with prior research (49–51). Furthermore, we observed an inverse correlation between physical exercise volume and resting-state *θ*(FP1 + FP2) power. Previous literature highlights that cognitively impaired older adults often exhibit reduced physical activity and prolonged sedentary behavior (52). Such physical inactivity may be associated with alterations in cerebrovascular structure and functional network connectivity, both of which are potentially linked to diminished inhibitory control and cognitive decline (53, 54). Conversely, exercise-related adaptations in the left dorsolateral prefrontal cortex (manifested as attenuated prefrontal *θ* activity), coupled with enhanced cerebral lactate availability, increased cerebral blood flow, and the up-regulation of brain-derived neurotrophic factor (BDNF), may collectively contribute to the improved neurocognitive outcomes observed in this population (55–57).

Our results highlight *θ*(FP1 + FP2) as a specific EEG indicator significantly associated with both cognitive function and inhibitory control in this demographic. The prefrontal cortex is well-established as a critical hub for executive functions, including attention, working memory, and emotional regulation (58). According to the Posterior–Anterior Shift in Aging (PASA) hypothesis (59), older adults often hyper-activate frontopolar regions to compensate for functional decrements in posterior brain areas during cognitive tasks. Within this network, the frontopolar cortex (Brodmann Area 10) exerts top-down control over multitasking, high-level inhibition, and goal-directed behaviors (60). Empirical evidence consistently shows that patients with mild cognitive impairment and Alzheimer’s disease exhibit a pronounced increase in resting-state slow-wave power (*θ* and *δ*) in anterior prefrontal regions (FP1, FP2), which correlates robustly with global cognitive severity (61, 62). Given that age-related inhibitory deficits are largely attributed to prefrontal deterioration (63), the elevated resting-state *θ* activity may reflect either the mobilization of compensatory mechanisms or the underlying degradation of neural networks. The significant negative correlations found in this study between *θ*(FP1 + FP2) power, inhibitory control, and cognitive function suggest that frontopolar *θ* power may serve as a sensitive neuroelectrophysiological signature for cognitive and executive decline. Importantly, *θ*(FP1 + FP2) in this study represents eyes-closed resting-state frontopolar *θ* power, not task-induced frontal-midline *θ* during active cognitive control (58). Thus, its negative association with cognitive and inhibitory control performance should not be interpreted as evidence that physical exercise directly suppresses task-related *θ* activity. Rather, lower resting-state frontopolar *θ* power may reflect reduced pathological slow-wave activity or greater neural efficiency in older adults with cognitive impairment (61, 62, 64), which should be further examined using combined resting-state and task-based EEG designs.

An exploratory finding of this study is that *θ*(FP1 + FP2) power and inhibitory control may constitute a statistical chain mediation pathway linking physical exercise volume with cognitive function. This pathway suggests a possible neurobehavioral association among physical exercise, resting-state frontopolar *θ* activity, inhibitory control, and global cognitive function. Consistent with previous evidence that physical activity is related to age-related cognitive health (65), higher physical exercise volume in the present study was associated with lower *θ*(FP1 + FP2) power, which may in turn be linked to better inhibitory control and cognitive performance. However, given the cross-sectional design and the elevated RMSEA, this pathway should be interpreted as a possible statistical pathway rather than evidence of a causal neurobiological mechanism. In parallel, the indirect pathway through inhibitory control was statistically significant, accounting for 21.31% of the total association. The indirect pathway through *θ*(FP1 + FP2) power was also significant, accounting for 13.11% of the total association. These findings suggest that the association between physical exercise volume and cognitive function may be partly explained by resting-state neural oscillatory activity and inhibitory control. Nevertheless, these mediation findings remain exploratory and require confirmation in longitudinal or intervention studies.

In conclusion, this observational study examined the complex interrelationships among physical exercise, inhibitory control, resting-state EEG, and cognitive function, identifying a multi-stage mediation pathway. These findings support the potential relevance of physical exercise in future geriatric cognitive intervention research. Future research should investigate the distinct neurobiological mechanisms driven by different exercise modalities and intensities, while considering the moderating effects of genetic and psychological individual differences.

### 4.1 Limitations

Several limitations warrant consideration. First, the sample was not clinically diagnosed and was drawn from only four senior care centers in Shanghai, introducing geographic and institutional selection biases that may limit generalizability. Second, physical exercise volume was assessed via subjective self-reports, which are susceptible to recall biases; future studies should integrate objective metrics like accelerometry. Third, the cross-sectional design inherently precludes causal inferences. Longitudinal cohorts and randomized controlled trials are essential to confirm directionality and establish evidence-based exercise dosage parameters. Fourth, despite applying *p*-value adjustments for multiple comparisons, residual risks of false-positive results cannot be entirely ruled out. Fifth, the use of a 16-channel EEG system limited spatial resolution. Although frontopolar electrodes (FP1/FP2) are highly sensitive to ocular artifacts, we mitigated this through rigorous ICA-based correction and strict amplitude thresholding; nevertheless, minor influences may persist. Sixth, depressive symptomatology—a highly prevalent confounder linked to both cognitive decline and physical inactivity—was not systematically evaluated and should be controlled for in future models (e.g., using the Geriatric Depression Scale). Seventh, the fixed block order of the Stroop task may have introduced practice or fatigue effects, and speed-accuracy trade-offs were not explicitly analyzed. Finally, the SEM showed an elevated RMSEA, indicating suboptimal absolute model fit; therefore, the robustness of this multi-mediation pathway requires further empirical validation.

## 5 Conclusion

Higher physical exercise levels were associated with better inhibitory control and cognitive function in older adults with cognitive impairment. *θ*(FP1 + FP2) power was identified as a shared EEG indicator associated with both inhibitory control and cognitive function. Exploratory covariate-adjusted SEM suggested that *θ*(FP1 + FP2) power and inhibitory control may statistically mediate the association between physical exercise and cognitive function. These findings may help inform future longitudinal and intervention studies targeting cognitive impairment in older adults.

## Data Availability

The datasets presented in this article are not readily available because the datasets generated and/or analyzed during the current study are not publicly deposited because they contain sensitive information from older adults with cognitive impairment and form part of an ongoing series of studies by the research team. De-identified data supporting the findings of this study are available from the corresponding author upon reasonable request for legitimate academic purposes, subject to approval by the research team and, where necessary, the relevant ethics committee, and under an appropriate data-use agreement. Requests to access the datasets should be directed to Tao Meng 20029305@lixin.edu.cn.
